# The Effects of CX_3_CR1 Deficiency and Irradiation on the Homing of Monocyte-Derived Cell Populations in the Mouse Eye

**DOI:** 10.1371/journal.pone.0068570

**Published:** 2013-07-03

**Authors:** Jelena M. Kezic, Paul G. McMenamin

**Affiliations:** 1 Department of Anatomy and Developmental Biology, Monash University, Clayton, Victoria, Australia; 2 Centre for Eye Research Australia, Department of Ophthalmology, University of Melbourne, Royal Victorian Eye and Ear Hospital, East Melbourne, Victoria, Australia; The University of Melbourne, Australia

## Abstract

This study examined whether CX_3_CR1 deficiency altered monocytic cell replenishment dynamics in ocular tissues in the context of radiation chimeras. Long-term effects of irradiation and effects of sublethal irradiation on ocular macrophages were also assessed. Bone marrow from BALB/c *Cx*_*3*_ *cr1* ^*+/gfp*^ or *Cx*_*3*_ *cr1* ^gfp/gfp^ mice was used to reconstitute full body irradiated WT mice and donor cell densities in the uveal tract were compared at 4 and 8 weeks post-transplantation. BALB/c and C57BL/6J chimeric mice were examined at 6 months of age to determine strain-related differences in microglial replenishment and radiation sensitivity. A separate cohort of mice were sublethally irradiated (5.5 Gy) and retinal tissue assessed 8 and 12 weeks later. CX_3_CR1 deficiency altered the early replenishment of monocytes in the posterior iris but not in the iris stroma, choroid or retina. In six month old chimeric mice, there were significantly higher GFP^+^ cell densities in the uveal tract when compared to non-irradiated 8-12 week old *Cx*_*3*_ *cr1* ^*+/gfp*^ mice. Additionally, MHC Class II expression was upregulated on hyalocytes and GFP^+^ cells in the peripheral retina and the repopulation of microglia appeared to be more rapid in C57BL/6J mice compared to BALB/c mice. Transient expression of MHC Class II was observed on retinal vasculature in sublethally irradiated mice. These data indicate CX_3_CR1-deficiency only slightly alters monocyte-derived cell replenishment in the murine uveal tract. Lethal irradiation leads to long-term increase in monocytic cell density in the uveal tract and retinal microglial activation, possibly as a sequelae to local irradiation induced injury. Microglial replenishment in this model appears to be strain dependent.

## Introduction

CX_3_CR1, the sole receptor for the chemokine CX_3_CL1, or fractalkine, is expressed by monocyte-derived cells including dendritic cells (DCs), natural killer cells and macrophages [1,2]. The differential expression of this receptor by tissue resident or ‘non-inflammatory’ macrophages (CX_3_CR1^high^ and CCR2^low^) and ‘inflammatory’ macrophages (CX_3_CR1^low^ CCR2^high^) [3] has been valuable in elucidating the role of CX_3_CR1 in different disease models [4–7]. In particular, the regulation of inflammation by CX_3_CL1/CX_3_CR1 signaling in central nervous system (CNS) tissues has been well established. In mouse experimental autoimmune encephalomyelitis (EAE), earlier disease onset and increased disease severity was observed in *Cx*_*3*_ *cr1* ^gfp/gfp^ mice [8], whilst the neutralization of CX_3_CL1 led to exaggerated LPS-induced neuroinflammation [9,10]. Similarly, CX_3_CR1 deficiency was associated with greater neuronal cell loss in mouse models of Parkinson’s disease and amyotrophic lateral sclerosis [9]. In the eye, data on the role of CX_3_CL1/CX_3_CR1 signaling have not provided a clear mechanism of action. Namely, whilst impairments in the dynamic behavior of retinal microglia in the resting state, and impaired microglial migration following laser injury in CX_3_CR1 deficient mice have been reported [11], CX_3_CR1 deficiency does not affect microglial responses to acute light damage [12] or proliferative neovascularization in a mouse model of retinopathy of prematurity [13]. Conflicting data also exist on the contribution of CX_3_CR1 to the pathogenesis of experimental autoimmune uveoretinitis (EAU) [14,15].

The effects of CX_3_CR1 deficiency on monocytic cell migration to ocular and peripheral tissues is largely dependent on the microenvironment of the destination tissue. Whilst the homing capacity of subpopulations of monocyte-derived DCs to epithelial surfaces of the small intestine [16], cornea [17] and olfactory epithelium [18] is impaired in the absence of CX_3_CR1, such receptor absence does not alter the normal distribution of macrophages and DCs in the uveal tract or retina of the mouse eye [19]. A recent study also demonstrated complete retinal microglial turnover following irradiation and bone marrow (BM) transfer was not CX_3_CR1 dependent, but rather, is regulated by CCL2/CCR2 signaling [20]. However, whether the early dynamics of monocyte-derived cell replenishment in the uveal tract and retina following lethal irradiation and BM transfer are altered with CX_3_CR1 deficiency in the donor cell population is unknown.

The homeostatic turnover of monocyte-derived cells has traditionally been studied using radiation BM chimeric models. In non-neural tissue we have reported almost complete repopulation of corneal, iris and choroidal macrophages eight weeks after whole-body irradiation and BM transplantation in the mouse, a rate which is considerably more rapid than in retina [21,22], where CNS microglial turnover has been reported to take 6 months in the mouse and up to a year in rats [23–25]. The more recent use of the parabiotic mouse model, which obviates the need for irradiation and BM transfer, has led to the idea that in the non-diseased, non-irradiated CNS, there is no homeostatic turnover of microglia, and as such, microglial progenitors exist within the CNS tissues and act as a reservoir for replenishment [26,27]. This new paradigm has led to the proposal that the turnover seen in radiation chimeras may in fact be due to a surge in BM-derived progenitors in the irradiated host when the host BM preparation is injected following myeloablation therapy, and that irradiation treatment can in itself act as a minor insult to the eye, thereby stimulating the turnover of BM-derived cells in ocular tissues [20,28]. Indeed, Chen et al (2012) have demonstrated DNA damage as well as low-grade chronic inflammation in the neural retina followed a single dose of whole-body γ-irradiation [20].

In the context of trying to understand the mechanisms of microglial activation or replenishment it is noteworthy that lethal irradiation and BM transfer, or even sublethal irradiation alone, has been shown to have protective effects in the *DBA/2J* mouse model of glaucoma [29,30]. Similarly, low-dose γ-irradiation has a protective effect on the survival of retinal ganglion cells in rodent models of NMDA toxicity and optic nerve crush [31]. Thus, determining the effects of lethal irradiation and BM transfer, as well as sublethal irradiation on the turnover of retinal microglia and retinal macrophages is timely if irradiation or BM therapy are to be considered as potential therapeutic applications for ocular diseases. In the present study, we report altered early replenishment rates of CX_3_CR1-deficient monocyte-derived cells to the posterior surface of the iris but not the iris stroma, choroid or the retina following BM transplantation. Additionally, whole-body irradiation led to a long-term increase in monocyte-derived cell density in the uveal tract and the activation of microglia in the retina suggesting a degree of low-grade inflammation. Previously unrecognized strain differences in the turnover of retinal microglia were also noted, with the replenishment rate of microglia appearing to be much faster in C57BL/6J mice compared to BALB/c mice. Sublethal irradiation resulted in the transient upregulation of MHC Class II expression on the retinal vasculature, but only minimal recruitment of BM-derived cells to the retina 8 and 12 weeks after irradiation treatment.

## Materials and Methods

### Experimental animals

Female C57BL/6J and BALB/c wild-type (WT) mice aged between 6–12 weeks were obtained from the Animal Resources Centre (Murdoch University, WA). *Cx*_*3*_ *cr1* ^*+/gfp*^ and *Cx*_*3*_ *cr1* ^*gfp/gfp*^ (CX_3_CR1-deficient) transgenic mice on BALB/c and C57BL/6J (wt/wt when screened for the *rd8* mutation in the *Crb1* gene) backgrounds were originally obtained from Dr Steffen Jung (Weizmann Institute of Science, Rehovot, Israel) [32] and were bred at the Animal Research Laboratory (Monash University, Clayton, VIC). *Cx*_*3*_ *cr1* ^*gfp/gfp*^ mice contain an enhanced green fluorescent protein (eGFP) encoding cassette knocked into the CX_3_CR1 gene that disrupts its expression but facilitates GFP expression under the control of the CX_3_CR1 promoter. *Cx*_*3*_ *cr1* ^*+/gfp*^ mice were generated by crossing *Cx*_*3*_ *cr1* ^*gfp/gfp*^ mice with WT mice. All mice were kept under pathogen free conditions in chaff-lined cages with individual ventilation in 12 hour day/night cycles. Food (Stockfeeders RM2 autoclaved rat and mouse diet) and water was supplied *ad libitum*. All procedures conformed to the ARVO statement for the use of animals in ophthalmic and vision research and were approved by the Monash University Animal Research Platform.

### Creation of bone marrow chimeras

Bone marrow (BM) chimeras were created using *Cx*_*3*_ *cr1* ^*+/gfp*^ and *Cx*_*3*_ *cr1* ^gfp/gfp^ mice as BM donors on either a BALB/c or C57BL/6J background. Recipient BALB/c or C57BL/6J WT mice were either a) full body irradiated (lethal) with two doses of 5.5 Gy 14h apart, or b) received a single irradiation dose of 5.5 Gy (sublethal). No head shielding was used for irradiation experiments. Donor *Cx*_*3*_ *cr1* ^*gfp/gfp*^ mice were euthanized and femurs and tibia harvested as previously described [33]. Briefly, following removal of the proximal and distal ends of the bone, the shafts were centrifuged at 10000 rpm for 30 seconds at 4°C. The pellet was resuspended in RPMI media (N6396; Sigma), centrifuged at 1200 rpm for 5 min at room temperature (RT), resuspended again and live cells counted by trypan blue exclusion. Recipient mice received an injection of 3-5 x 10^6^ BM cells (in 150 µl) into the lateral tail vein (approximately 2-3 h after the second dose of irradiation). Chimeric animals were sacrificed at 4 and 8 weeks post-transplantation (n= 4 mice per time point) for collection of ocular tissues. A separate cohort of *Cx*_*3*_ *cr1* ^*+/gfp*^ →WT chimeric mice were sacrificed at 6 months post irradiation (n = 6) and BM transfer to determine the long-term effects of irradiation, as well as strain differences in microglial replenishment. Mice in sublethal irradiation experiments were sacrificed at 8 weeks (n= 6 per group) and 12 weeks (n= 6 per group) after treatment.

### Tissue collection and processing for immunofluorescence staining

Mice were perfusion-fixed using 4% paraformaldehyde as previously described and from each eye, the iris, ciliary body, choroid and retina were dissected free and cut into quadrants as documented [34]. Donor BM-derived monocytic cells from *Cx*_*3*_ *cr1* ^*+/gfp*^ and *Cx*_*3*_ *cr1* ^gfp/gfp^ mice are GFP^+^ and thus appear green upon fluorescent microscopic examination. To identify host-derived cells (GFP^-^) and to further characterize donor-derived GFP^+^ cells, we performed immunofluorescence staining using a range of antibodies specific for macrophages. Tissue pieces were incubated in 20 mM EDTA tetrasodium at 37°C for 30 min, then incubated with a 0.3% solution of Triton-X in PBS with 3% bovine serum albumin at RT for 60 min. Tissues were treated at 4°C overnight with the monoclonal antibodies anti-MHC class II (M5/114; BD Pharmingen, San Diego, CA, USA) and anti-CD11b (Integrin expressed on cell surface of monocytes; BD Pharmingen) and the polyclonal antibody anti-Iba-1 (Ionized calcium binding adaptor molecule-1, microglia/macrophage specific marker; Wako Pure Chemical Industries Ltd., Osaka, Japan) as well as isotype controls (IgG2a and IgG2b, BD Pharmingen). Tissues were then treated with biotinylated goat anti-rat IgG (Amersham Biosciences, Piscataway, NJ, USA) at RT for 60 min, washed with PBS and incubated with streptavidin-Cy3 (Jackson ImmunoResearch Laboratories, West Grove, PA, USA) at RT for 60 min. Hoescht (Molecular Probes - Invitrogen, Mulgrave, VIC, Australia) was added at RT for 8 min as a nuclear stain. Stained whole mount tissues were mounted onto slides (retinae were mounted with vitreous side face up) using aqueous mounting medium (Immunomount; Thermo Shandon, Waltham, MA, USA) and coverslipped.

### Examination of whole mount tissue and quantitative analysis

Stained specimens were examined by epifluorescence (Olympus, Tokyo, Japan; DMRBE, Leica, Australia) and confocal microscopy (Leica TCS SP2). Images of entire tissue whole mount preparations were produced by performing Z-scans of the tissue from the internal to external aspect at increments ranging from 0.6 µm to 1 µm. Adobe Photoshop (Version 7.0) was used to perform final image processing. Donor GFP^+^ cell numbers and MHC Class II expression on GFP^+^ cells were counted in a masked fashion from a minimum of six randomly selected areas of each tissue and a mean cell density (per mm^2^) or percentage was calculated (Image ProPlus; Version 5.1).

### Statistical analysis

**Figure 1:**
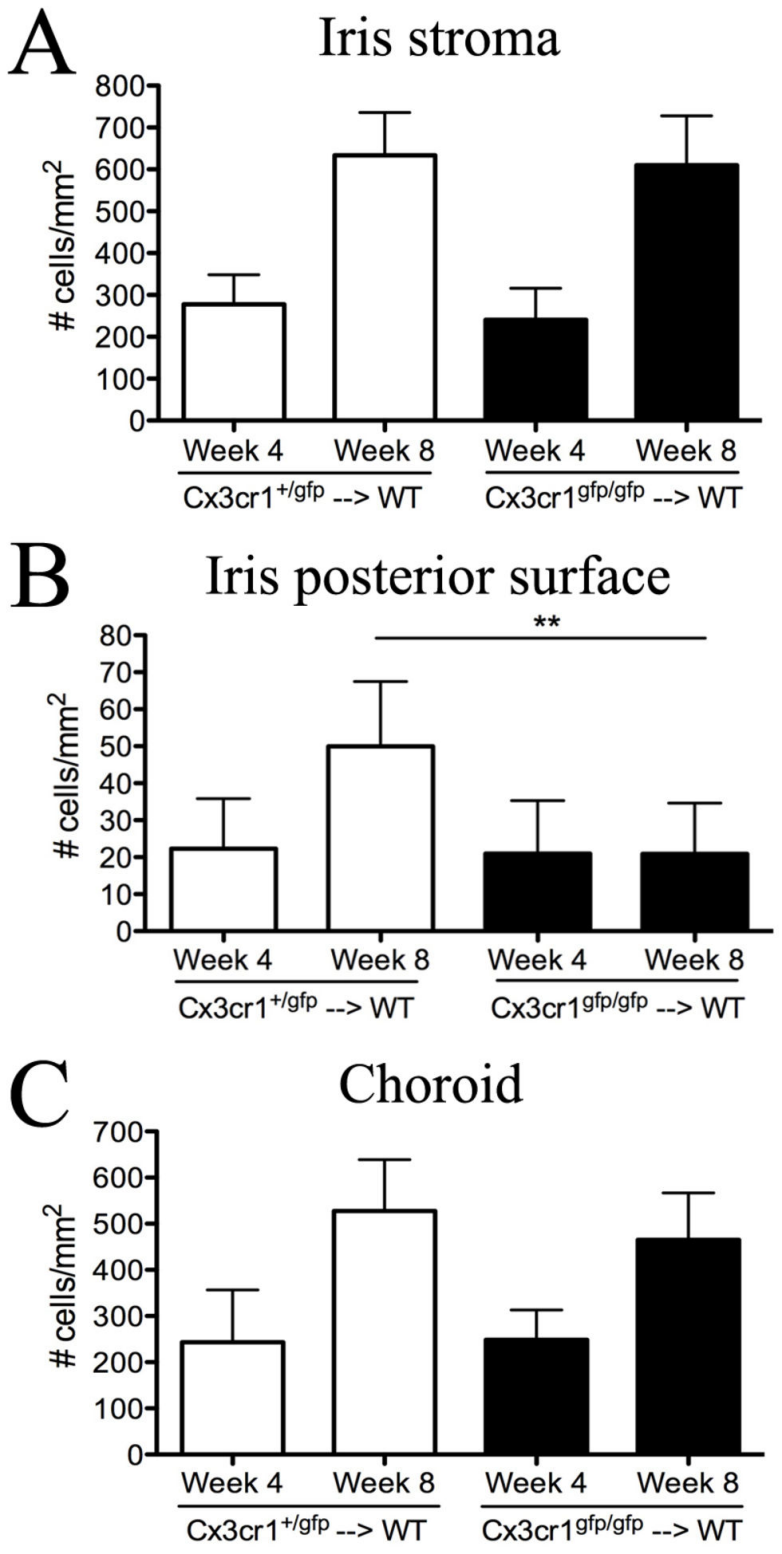
CX_3_CR1 deficiency alters the replenishment rate of monocyte-derived cells in the uveal tract of BALB/c mice. The density of GFP^+^ donor cells in the iris stroma (A), iris posterior surface (B) and choroid (C) in *Cx*_*3*_ *cr1* ^*+/gfp*^ → WT and *Cx*_*3*_ *cr1* ^*gfp/gfp*^ → WT chimeric mice at 4 weeks and 8 weeks post BM transfer. Data were analyzed by two-way ANOVA with Bonferroni post-hoc test. ** P < 0.01. n= 4 mice per group.

Data are presented as mean ± standard deviation. Data were analyzed using two-Way ANOVA with Bonferroni post-hoc test (Figure 1) or unpaired student’s t-test (Prism; Graph Pad Software, San Diego, CA).

## Results

### CX_3_CR1 deficiency has a minimal effect on the early replenishment of monocyte-derived cells in the uveal tract of BALB/c mice

We have previously shown that the absence of CX_3_CR1 does not affect the final density or distribution of ocular macrophages in the uveal tract [19]. Further, using conventional bone marrow (BM) chimeric mice whereby BM from *Cx*_*3*_ *cr1* ^+*/gfp*^ mice was used to reconstitute whole body irradiated BALB/c WT mice (*Cx*_*3*_ *cr1* ^*+/gfp*^ → WT), we demonstrated that donor-derived GFP^+^ cells begin to repopulate the uveal tract tissues as early as 2 weeks post transplantation, with almost complete replenishment at 8 weeks [33]. Here, we set out to build on these data and examine the temporal sequence of this monocytic cell turnover in order to determine whether CX_3_CR1 deficiency alters the rate of repopulation of monocytic cells in these tissues. The replenishment of GFP^+^ monocyte-derived cells in the uveal tract was assessed in BALB/c chimeric mice that had received lethal irradiation and BM transfer from either *Cx*_*3*_ *cr1* ^*+/gfp*^ (*Cx*_*3*_ *cr1* ^*+/gfp*^ → WT) or *Cx*_*3*_ *cr1* ^*gfp/gfp*^ (*Cx*_*3*_ *cr1* ^gfp/gfp^ → WT) donor mice. There were no significant differences in the repopulation of GFP^+^ donor cells in the iris stroma (Figure 1A) and choroid (Figure 1C) of chimeric mice that had received either *Cx*_*3*_ *cr1* ^*+/gfp*^ or *Cx*_*3*_ *cr1* ^*gfp/gfp*^ BM at both 4 weeks and 8 weeks post BM transfer. However, the emigration of GFP^+^ cells to the posterior surface of the iris (Figure 1B) was significantly altered by CX_3_CR1 deficiency on BM cells at week 8 (*P* < 0.01).

### CX_3_CR1-deficiency does not affect the early replenishment dynamics of retinal microglia or vitreal hyalocytes in BALB/c mice

**Figure 2:**
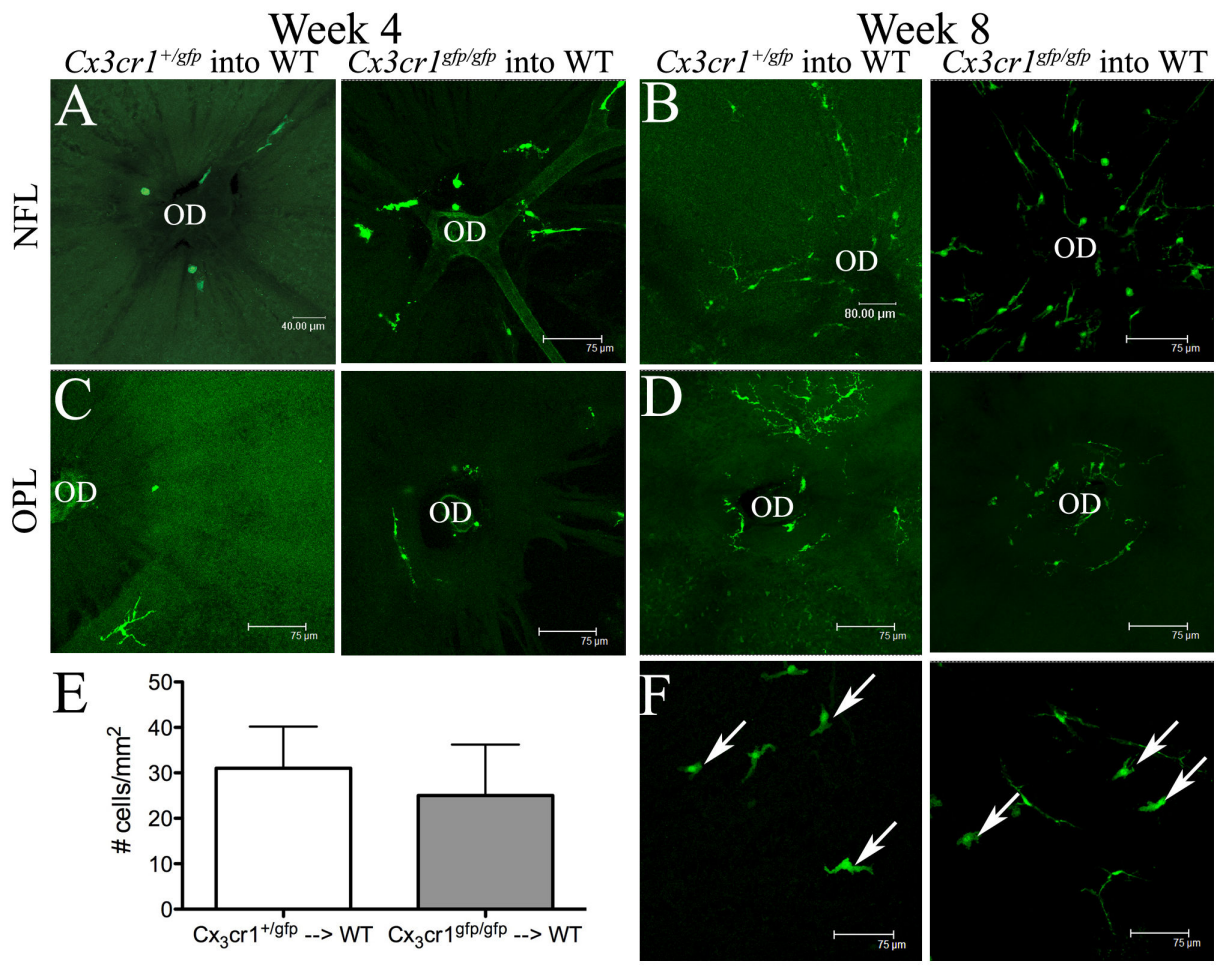
CX_3_CR1 deficiency does not influence the replenishment of retinal microglia or vitreal hyalocytes in BALB/c mice. At 4 weeks post BM transfer, donor GFP^+^ cells were present in small numbers surrounding the optic disc (OD) at the level of the NFL (A), inner plexiform layer (not shown) and OPL (C) of both *Cx*_*3*_ *cr1* ^+*/gfp*^ → WT and *Cx*_*3*_ *cr1* ^gfp/gfp^ → WT chimeric mice. At week 8, the proportion of GFP^+^ cells appeared to be similar in *Cx*_*3*_ *cr1* ^+*/gfp*^ → WT (B, D; left) and *Cx*_*3*_ *cr1* ^gfp/gfp^ → WT chimeric mice (B, D; right). The cell density of donor GFP^+^ vitreal hyalocytes in *Cx*_*3*_ *cr1* ^+*/gfp*^ → WT and *Cx*_*3*_ *cr1* ^gfp/gfp^ → WT chimeric mice was similar at 8 weeks post BM transfer (E; *P* = 0.12). Examples of hyalocytes indicated by white arrows (F). n= 4 mice per group. NFL, nerve fiber layer; OPL, outer plexiform layer.

Previous studies in C57BL/6 mice [24] have demonstrated the complete replenishment of retinal microglia by 6 months after whole body irradiation and BM transfer and more recently the same group reported that microglial turnover is highly dependent on CCL2/CCR2 signaling [20]. Our group have shown in BALB/c mice that entry of donor GFP^+^ myeloid derived cells commences and is restricted to the optic disc region of the retina as early as 4 weeks post myeloablation and BM transfer [33]. In the present study, we wished to examine whether CX_3_CR1 deficiency altered the timing of commencement of such donor cell entry into the retina in BALB/c chimeric mice and get a clearer picture of the temporal pattern of these early replenishment events. Qualitative assessment of donor-derived GFP^+^ cells in the nerve fiber layer (NFL; Figure 2A, B), inner plexiform layer (data not shown) and outer plexiform layer (OPL; Figure 2C, D) of retinal whole mounts revealed no striking differences in the proportion of GFP^+^ BM donor cells entering the retina at the region of the optic disc of *Cx*_*3*_ *cr1* ^*+/gfp*^ → WT and *Cx*_*3*_ *cr1* ^*gfp/gfp*^ → WT chimeric mice at either 4 weeks (Figure 2A, C) or 8 weeks (Figure 2B, D) post transplantation. Hyalocytes are easily distinguished from retinal microglia due to their pleiomorphic rather than ramified morphology as well as their vitread location in retinal whole mount preparations (Figure 2F; arrows). At 4 weeks, donor-derived GFP^+^ hyalocytes were absent from the retina of both *Cx*_*3*_ *cr1* ^*+/gfp*^ → WT and *Cx*_*3*_ *cr1* ^*gfp/gfp*^ → WT chimeric mice (data not shown), whilst at 8 weeks post transplantation, the density of GFP^+^ hyalocytes was not significantly different in *Cx*_*3*_ *cr1* ^*+/gfp*^ → WT and *Cx*_*3*_ *cr1* ^*gfp/gfp*^ → WT chimeric mice (Figure 2E, P = 0.12; F).

### The long-term effects of whole-body irradiation and BM transplantation on normal cell density and phenotype of monocyte-derived cells in the uveal tract

**Figure 3:**
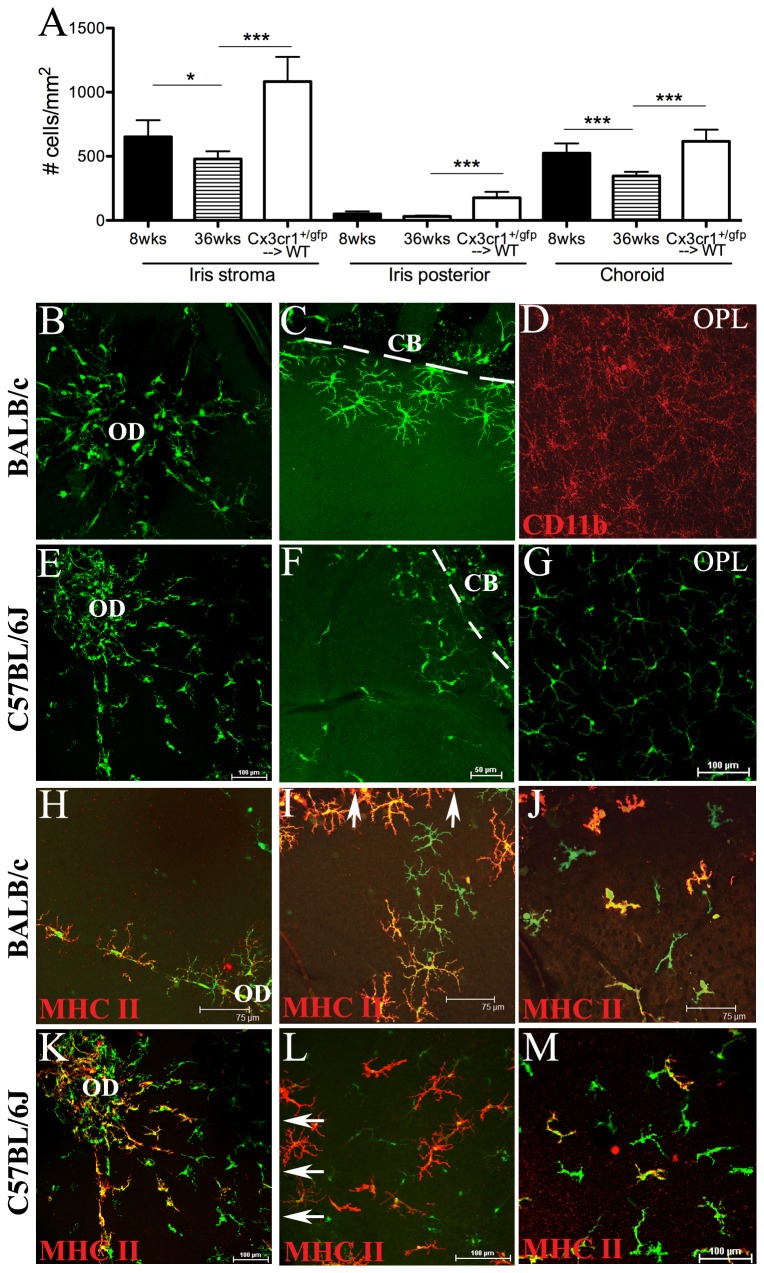
Long-term effects of lethal irradiation and BM transplantation on normal monocyte-derived cell density and phenotype in ocular tissues. Donor GFP^+^ cell densities in the iris and choroid of *Cx*_*3*_ *cr1* ^*+/gfp*^ → WT BALB/c chimeric mice 6 months after BM reconstitution (A). There were significantly higher numbers of GFP^+^ cells in all uveal tract tissues of 6 month old chimeric mice (*Cx*_*3*_ *cr1* ^*+/gfp*^ → WT) compared to 8 week old (8wks) and 36 week old (36 wks) non-irradiated naïve *Cx*_*3*_ *cr1* ^*+/gfp*^ mice (A). * P < 0.05, *** P < 0.0005. Confocal microscopic images of retinal whole mounts from 6 month old BALB/c (B–D, H–J) and C57BL/6J (E–G, K–M) *Cx*_*3*_ *cr1* ^*+/gfp*^ → WT chimeric mice. Donor GFP^+^ cells migrating into the BALB/c retina from the optic disc region (B; OD) and at the retinal periphery (C; broken white line indicates border between peripheral retina and ciliary body). Host derived GFP^-^ CD11b^+^ microglia were present throughout the OPL of the retinal parenchyma (D). GFP^+^ cells replenishing retinal tissue of C57BL/6J chimeric mice at the optic disc region (E) and periphery (F; broken white line indicates border between peripheral retina and ciliary body). Donor-derived GFP^+^ microglia in the OPL of C57BL/6J chimeric mice (G). Perivascular GFP^+^ cells at the optic disc region expressed MHC Class II in BALB/c (H; red) and C57BL/6J (K; red) chimeric mice. A population of GFP^+^ cells entering the retina at the periphery were MHC Class II^+^ in BALB/c (I; red) and C57BL/6J (L; red) chimeric mice. I, L; arrows point towards ciliary body at the retinal periphery. A subpopulation of GFP^+^ vitreal hyalocytes were MHC Class II^+^ in BALB/c (J) and C57BL/6J (M) chimeric mice. n= 6 mice per group. CB, ciliary body; OPL, outer plexiform layer.

The aim of the next part of this study was to assess the long-term effects of unshielded irradiation on the monocyte-derived cell populations in the uveal tract tissues. To do this, we assessed ocular tissues of *Cx*_*3*_ *cr1* ^*+/gfp*^ → WT BALB/c mice at 24 weeks after transplantation, when mice are approximately 6 months of age. Since the above data showed minimal alterations to early cell replenishment, and a previous study [19] suggested no major differences in the final density or distribution of donor myeloid cells in the uveal tract, we chose only to perform experiments using *Cx*_*3*_ *cr1* ^*+/gfp*^ → WT chimeric mice. The number of GFP^+^ cells were counted and compared to the normal density of eGFP^+^ cells in the uveal tract of 8 week and 36 week old non-irradiated naïve *Cx*_*3*_ *cr1* ^*+/gfp*^ mice. There were significantly higher numbers of GFP^+^ donor cells in the iris and choroid of BM chimeric mice than in naïve WT mice (Figure 3A) which indicates irradiation and bone marrow transfer enhances monocytic recruitment to these tissues suggesting some degree of tissue injury. Interestingly, a previously unrecognized age effect was noted in non-irradiated naïve *Cx*_*3*_ *cr1* ^*+/gfp*^ mice in which the density of GFP^+^ cells in the iris stroma and choroid were lower at 36 weeks when compared to 8 week old mice (Figure 3A).

### Strain differences in radiation sensitivity in the retina

We next chose to examine the long-term effects of unshielded lethal irradiation and BM transfer in the retina of both C57BL/6J and BALB/c mice, and assess any strain-related differences in radiation sensitivity. In BALB/c *Cx*_*3*_ *cr1* ^*+/gfp*^ → WT chimeric mice (Figure 3B–D), GFP^+^ donor cells were located only in the juxtapapillary region around the optic disc (Figure 3B) and the peripheral margin (junction between the peripheral retina and ciliary body) (Figure 3C). Host derived microglia (GFP^-^ CD11b^+^) were evident throughout the inner plexiform (data not shown) and outer plexiform (Figure 3D) layers of the retinal parenchyma. In contrast, in C57BL/6J *Cx*_*3*_ *cr1* ^*+/gfp*^ → WT chimeric mice, donor-derived GFP^+^ cells were more widespread throughout the retinal parenchyma, being located around the optic disc region (Figure 3E), at the peripheral margin (Figure 3F) and also in the inner plexiform (data not shown) and outer plexiform (Figure 3G) layers. These data indicate newly derived GFP^+^ microglia appeared to more rapidly populate the retina in C57BL/6J WT chimeric mice compared to BALB/c chimeric mice at 6 months. These observations are akin to what has previously been reported in C57BL/6 BM chimeric mice [24]. As expected, perivascular macrophages along the inner retinal vessels expressed MHC Class II in both BALB/c (Figure 3H) and C57BL/6J (Figure 3K) chimeric mice. Microglia in the naive murine retina are generally MHC Class II^-^ with the exception of perivascular macrophages and a minor subpopulation surrounding the optic disc [35]. Interestingly, a large proportion of donor GFP^+^ cells at the peripheral margin were MHC Class II^+^ (Figure 3I, L; arrows point towards ciliary body at the retinal periphery), indicative of an increase in the expression of this activation marker following irradiation and BM transfer. In BALB/c chimeric mice, 85% of GFP^+^ cells in this region were MHC Class II^+^ (85 ± 14.82), which was significantly higher than the 57% of GFP^+^ cells at the peripheral margin that were MHC Class II^+^ (57.05 ± 2.002) in C57BL/6J mice (*P* = 0.02). MHC Class II was also evident on GFP^+^ hyalocytes, being similarly expressed in BALB/c (Figure 3J; 62% of GFP^+^ hyalocytes) and C57BL/6J (Figure 3M; 74% of GFP^+^ hyalocytes) chimeric mice (*P* = 0.07), indicative of activation of a subpopulation of these cells. In the naïve mouse retina, hyalocytes are normally MHC Class II^low^ or MHC Class II^-^ [36]. The density of hyalocytes in chimeric mice at 6 months post BM reconstitution was 72.35 ± 5.662 cells/mm^2^, considerably higher when compared to the density of hyalocytes in naïve WT mice reported in previous studies [36].

### The effects of sublethal irradiation on the naïve retina

**Figure 4:**
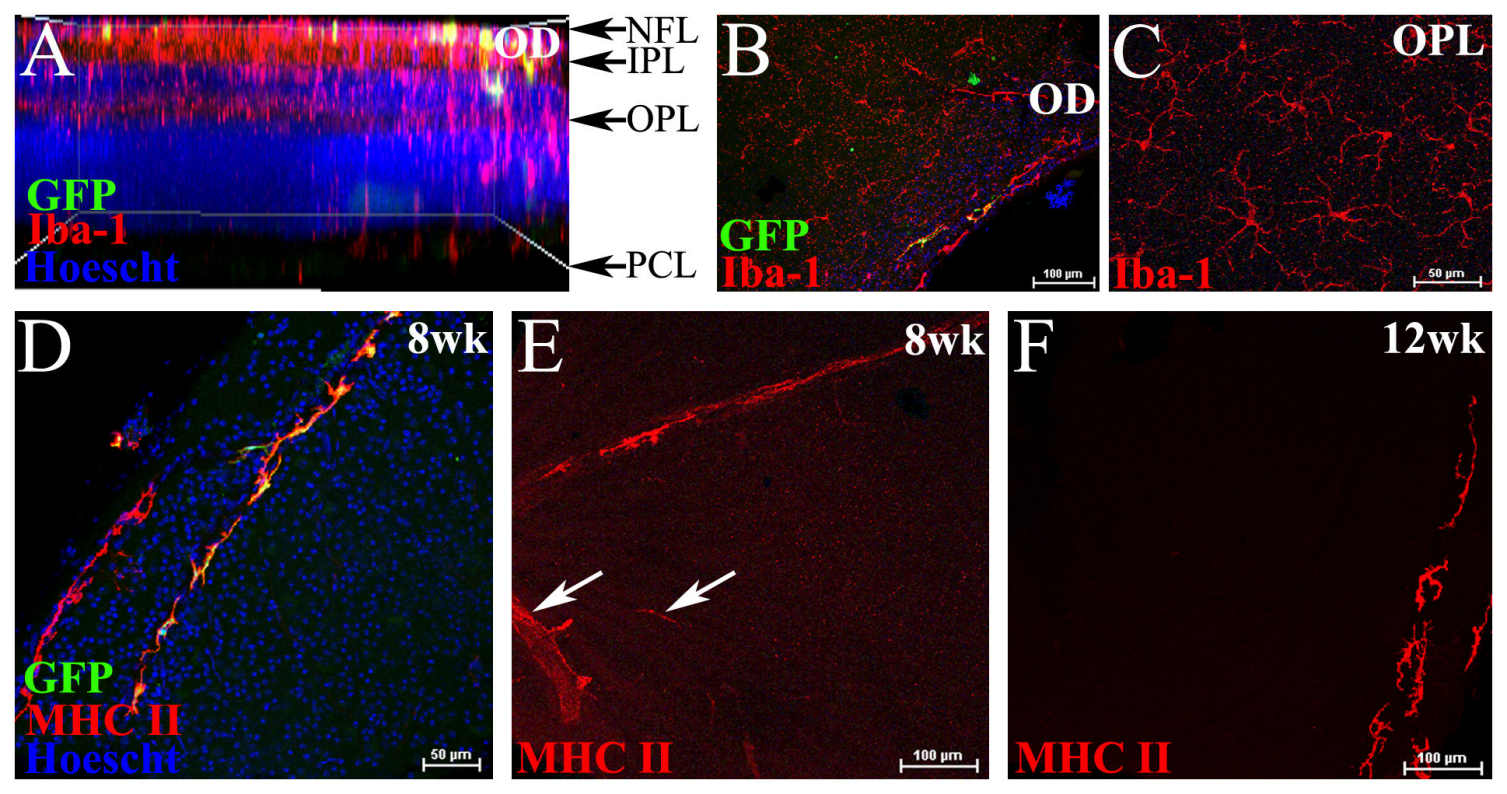
The acute effects of sublethal irradiation on the retina. Confocal microscopic analysis of retinal whole mounts from C57BL/6J mice that had received sublethal irradiation (5.5 Gy) plus *Cx*_*3*_ *cr1* ^*+/gfp*^ BM transfer. Whole retinal scan viewed in side profile showing few GFP^+^ donor cells in the NFL and IPL of the retina at 8 weeks post-irradiation and BM transfer (A). *En face* image at the optic disc (OD) region showing few GFP^+^ cells in chimeric mice at 8 weeks (B). Host derived GFP^-^ Iba-1^+^ microglia in the OPL (C). Donor derived GFP^+^ cells at the peripheral retina were mainly perivascular and expressed MHC Class II (D). MHC Class II was expressed on some of the inner retinal vasculature 8 weeks after sublethal irradiation (E, white arrows), but not at 12 weeks, where only perivascular cells at the periphery were MHC Class II^+^ (F). n= 6 mice per group. NFL, nerve fibre layer; IPL, inner plexiform layer; OPL, outer plexiform layer; OD, optic disc; PCL, photoreceptor cell layer.

Sublethal irradiation as a protective treatment in mouse models of glaucoma [30,37] has raised awareness of the potential of this therapeutic option. In light of this we wished to determine whether a single sublethal dose of unshielded irradiation (5.5Gy) would induce low grade inflammation as observed in lethally irradiated BM chimeric mice. As a lead up to future experiments that will investigate the protective effects of sublethal irradiation in models of retinal injury, eight week old C57BL/6J mice received sublethal irradiation (5.5 Gy) plus BM transfer (without head shielding) and retinal tissue was assessed 8 and 12 weeks later. Analysis revealed minimal influx of donor-derived cells in the retina of sublethally irradiated chimeric mice, with few GFP^+^ cells entering the optic disc region at 8 weeks (Figure 4A, B) and 12 weeks (data not shown). Host-derived GFP^-^ Iba-1^+^ microglia were present throughout the inner plexiform (not shown) and outer plexiform layers (Figure 4A, C), further indicative of minimal donor cell entry at this stage. MHC Class II was expressed on both GFP^-^ and GFP^+^ perivascular cells in the peripheral retina (Figure 4D), as well as on some inner retinal vascular endothelium in the central retina at 8 weeks post-irradiation treatment (Figure 4E; white arrows). This expression of MHC Class II on the retinal vasculature was transient, being absent at 12 weeks, with MHC Class II expression only evident on perivascular cells (Figure 4F; peripheral retina).

## Discussion

The homing or recruitment of immunocompetent cells to the eye and other tissues during normal and pathological conditions is regulated in part by chemokines, with the dysregulation of chemokine receptor expression having been implicated in the development of a number of inflammatory conditions including autoimmune disease [5,7,8,38,39]. In particular, the CX_3_CL1-CX_3_CR1 pathway is a key mediator of leukocyte chemotaxis and adhesion in human diseases such as atopic dermatitis [40], and in the animal models collagen-induced arthritis [41], experimental autoimmune myositis [42] and experimental autoimmune enecephalomyelitis [43]. In the present study, we explored whether the absence of CX_3_CR1 influenced the rate at which hematogenous monocyte-derived cells replenished the resident macrophages/myeloid cells of the retina and uveal tract in the context of experimentally induced conditions such as host strain and radiation doses. Alterations to the rate of monocytic cell homing to different tissue microenvironments have implications for disease dynamics and responses to inflammation. In particular, chemokine deficiency or impairments in chemokine signaling alter the dynamics of monocytic cell responses to inflammation or tissue injury [44,45], an important factor when determining the timing of therapeutic interventions in models of disease.

In the present study, we demonstrate minimal effect from CX_3_CR1 deficiency on the early replenishment of retinal microglia and the homing of monocytic cells to the uveal tract. Our data showing CX_3_CR1 associated impairment of monocytic cell recruitment to the posterior surface of the iris, an epithelial surface, are unsurprising. Previous studies in CX_3_CR1 deficient mice have demonstrated impaired migration of dendritic cells to epithelial tissues in the cornea [17], olfactory tract [18] and gut [16]. These findings suggest that the effects of CX_3_CR1 deficiency on monocytic cell migration in the context of radiation chimeras may be specific to epithelial tissues. Interestingly, Łyszkiewicz et al [46] have previously shown that during conditions of irradiation-induced inflammation, CX_3_CR1 signaling appears to play a minor role in the development of macrophages and dendritic cells. However, when redundancy was overcome by using adoptive transfer of CX_3_CR1 deficient cells directly into the spleen and thymus, this receptor promoted the development of these myeloid cells in homeostatic conditions. This highlights one potential caveat of using *Cx*_*3*_ *cr1* ^gfp/gfp^ mice to create BM chimeras in order to investigate the role of CX_3_CR1 deficiency in normal monocytic cell recruitment.

Despite their limitations, BM chimeric mice have served as useful models to determine the homing or recruitment of cells to various tissues in both homeostatic conditions and during disease. Specifically, such models have been used extensively to facilitate the study of resident versus hematogenous cell contribution to disease pathogenesis in both the eye and brain, such as during posterior uveoretinitis [15], brain ischemia [47], and light-induced photoreceptor damage [48]. However, the more recent use of parabiotic mouse models has brought to light further limitations involved in using BM chimeras to study normal monocytic cell turnover. Indeed, it is now well recognized that unlike non-neural peripheral tissues, there is limited cell turnover of CNS microglia in the non-diseased or uninjured state and thus, increased turnover of microglia in the brain and retina is likely indicative of activation that occurs in a number of pathological conditions [26,28]. In support of these recent paradigm shifts in our understanding of neural microglia turnover, Müther et al. (2010) demonstrated that donor-derived GFP^+^ cells infiltrate the peripheral retina of BM chimeric mice only in those that had not been head-shielded at the time of irradiation [28]. London et al. noted that GFP^+^ monocytes were not detected in the retina of BM chimeric mice that had been head-shielded during irradiation [49], further supporting the proposal that the irradiation used in myeloablation studies, in the absence of head shielding, produces some degree of tissue injury and consequent “wound healing” response in the eye. In the present study, although our data shed light on the influence of CX_3_CR1 deficiency on monocytic cell recruitment to ocular tissues, as well as outlining strain dependent differences in microglia recruitment following irradiation and BM transfer, these findings likely do not reflect normal monocyte homing to the retina in the absence of pathology or inflammation. Indeed, our data suggest the presence of low-grade inflammation even after sublethal irradiation and BM transfer, which is in line with a recent study investigating the effects of whole-body γ-irradiation in the retina [20].

We report almost complete replenishment of retinal microglia in the C57BL/6J mouse 6 months after lethal irradiation and BM transfer in the absence of head shielding. Intriguingly, there was limited cell replenishment in the BALB/c mouse at 6 months post BM reconstitution, indicative of strain related differences in radiation sensitivity and thus, microglial recruitment to the retina in the unshielded eye. Variations in the response to radiation exposure between inbred mouse strains has been well established. Differences in susceptibility to DNA damage and cell death, as well as differences in tissue responses and repair mechanisms, have been previously reported as the underlying mechanisms [50–52]. For example, greater radiation-induced DNA damage occurs in BALB/c mice, a radiation-sensitive strain, when compared to DNA damage in C57BL/6J mice, a radiation resistant strain in this context [53]. It is therefore interesting that in the present study, microglial turnover is much more rapid in C57BL/6J host mice when compared to BALB/c mice. Strain related differences in immune cell populations in the eye, which may be related to radiation sensitivity and disease susceptibility, have indeed been reported. For example, Xu et al (2007) demonstrated the presence of 33D1^+^ MHC Class II^high^ dendritic cell (DC) populations in the naïve C57BL/6 mouse eye in retinal locations corresponding to the earliest regions of inflammation during EAU, namely the peripheral margin and juxtapapillary region, whereas in BALB/c mice, a higher number of 33D1^+^ cells were identified in the same retinal regions that were MHC Class II^low/-^ [35]. The presence of retinal DCs around vulnerable regions of the retina may be related to blood retinal barrier integrity, with higher numbers of DCs correlating with increased resistance to EAU [54,55]. Thus, the faster rate of replenishment of retinal microglia in C57BL/6J mice when compared to BALB/c mice could reflect a more compromised blood retinal barrier in response to un-shielded whole body irradiation.

Another effect of un-shielded whole body irradiation on retinal tissue observed in the present study was the higher density of hyalocytes in chimeric mice at 6 months post BM reconstitution (72.35 ± 5.662 cells/mm^2^) when compared to the density of hyalocytes in 7 week old (10.7 ± 1.6 cells/mm^2^) and 120 week old naive WT mice (48.1 ± 5.6 cells/mm^2^) [36]. Similarly, cell density was higher in the iris stroma and choroid of 6 month old lethally irradiated mice when compared to age-matched controls that had not undergone irradiation. These data may be indicative of enhanced recruitment of blood monocyte progenitor cells to the ocular tissues following full-body “lethal” irradiation without head shielding. In contrast, we noted a lower density of GFP^+^ cells in the iris stroma and choroid of non-irradiated naïve *Cx*_*3*_ *cr1* ^*+/gfp*^ mice at 36 weeks of age when compared to 8 week old mice. These data are surprising as they differ to the reported increase in macrophage number in the subretinal space with aging, which has been hypothesized to be the result of chemokine receptor deficiencies on monocytic cells [56–58]. In addition to the accumulation of subretinal macrophages in normal aging the presence of these cells, which are normally absent from the subretinal space, are regarded as indicative of pathological changes or retinal inflammation [59–61]. Thus, the significance of decreased macrophage numbers in the uveal tract tissues of older mice observed in the present study requires further investigation.

Data are now emerging on the long-term effects of whole body irradiation on retinal tissue integrity. Very recently, Chen et al. (2012) demonstrated para-inflammatory like changes of at least 5 months duration in the retina, following unshielded whole-body irradiation [20]. Similarly, others have shown radiation-induced changes including neuronal cell apoptosis 4 months after irradiation treatment [28]. In the present study, we observed significantly higher densities of vitreal hyalocytes, and monocyte derived cells in the iris and choroid 6 months after irradiation and BM transplantation when compared to age matched non-irradiated mice. Additionally, our assessment of the long-term effects of whole body irradiation in the retina revealed para-inflammatory like changes which included the upregulation of MHC Class II expression on microglia and vitreal hyalocytes, indicative of activation of these cells. Collectively, these findings lead us to conclude irradiation-induced changes in the eye and retina should be considered when investigating disease mechanisms using BM chimeric models that do not use head shielding. Indeed, a limitation of the present study is that we did not perform experiments with head shielding, which would have allowed a direct comparison to our data from unshielded BM chimeric studies.

Despite the long-term effects of unshielded irradiation treatment on ocular tissues, various combinations of irradiation and BM transfer, irradiation alone, or even BM transplantation without myeloablation treatment have been used as a putative therapeutic approach in rodent models of retinal injury. High dose gamma (γ)-irradiation and syngeneic bone marrow transfer have been reported to prevent the development of naturally occurring hereditary glaucoma in the *DBA/2J* mouse, with protection maintained for up to 14 months of age [29]. Interestingly, further studies using low dose irradiation without BM transfer demonstrated that the protective effects of irradiation treatment on the development of glaucoma were largely due to a reduction in monocytic cell migration into the eye and reduced microglial activation [30,37]. Low-dose γ-irradiation without BM transfer was also reported to improve the survival of RGC in rodent models of NMDA toxicity and optic nerve crush [31]. In the present study, transient low-grade inflammation, as manifest by MHC Class II expression on the inner retinal vasculature, was detected in the retina 8 weeks after sublethal irradiation, however, this effect had diminished by 12 weeks. The long-term effects of sublethal irradiation on ocular tissue integrity, as well as further understanding the underlying mechanisms of the protective effects of irradiation treatment, will be important to establish whether irradiation is to be considered as a viable therapeutic approach for age-related ocular diseases such as glaucoma. Additionally, whether irradiation treatment with head shielding produces similar protective effects in the eye is yet to be determined and warrants further studies.
